# Active Hippocampal Networks Undergo Spontaneous Synaptic Modification

**DOI:** 10.1371/journal.pone.0001250

**Published:** 2007-11-28

**Authors:** Masako Tsukamoto-Yasui, Takuya Sasaki, Wataru Matsumoto, Ayako Hasegawa, Takeshi Toyoda, Atsushi Usami, Yuichi Kubota, Taku Ochiai, Tomokatsu Hori, Norio Matsuki, Yuji Ikegaya

**Affiliations:** 1 Laboratory of Chemical Pharmacology, Graduate School of Pharmaceutical Sciences, The University of Tokyo, Tokyo, Japan; 2 Department of Neurosurgery, Tokyo Women's Medical University, Shinjuku-ku, Tokyo, Japan; 3 Precursory Research for Embryonic Science and Technology (PRESTO), Japan Science and Technology Agency, Tokyo, Japan; Emory University, United States of America

## Abstract

The brain is self-writable; as the brain voluntarily adapts itself to a changing environment, the neural circuitry rearranges its functional connectivity by referring to its own activity. How the internal activity modifies synaptic weights is largely unknown, however. Here we report that spontaneous activity causes complex reorganization of synaptic connectivity without any external (or artificial) stimuli. Under physiologically relevant ionic conditions, CA3 pyramidal cells in hippocampal slices displayed spontaneous spikes with bistable slow oscillations of membrane potential, alternating between the so-called UP and DOWN states. The generation of slow oscillations did not require fast synaptic transmission, but their patterns were coordinated by local circuit activity. In the course of generating spontaneous activity, individual neurons acquired bidirectional long-lasting synaptic modification. The spontaneous synaptic plasticity depended on a rise in intracellular calcium concentrations of postsynaptic cells, but not on NMDA receptor activity. The direction and amount of the plasticity varied depending on slow oscillation patterns and synapse locations, and thus, they were diverse in a network. Once this global synaptic refinement occurred, the same neurons now displayed different patterns of spontaneous activity, which in turn exhibited different levels of synaptic plasticity. Thus, active networks continuously update their internal states through ongoing synaptic plasticity. With computational simulations, we suggest that with this slow oscillation-induced plasticity, a recurrent network converges on a more specific state, compared to that with spike timing-dependent plasticity alone.

## Introduction

Experience-dependent synaptic plasticity is a fundamental feature of neural networks involved in storing information [1]–[3]. Determining the rules that govern synaptic plasticity, therefore, is essential for understanding brain function. Several types of patterned activity are shown to efficiently induce synaptic plasticity [4]–[11], but in these experiments, artificial stimulation protocols were used to produce synaptic plasticity. In the brain, synaptic strength is modified by its internal activity through inherently defined rules, yet little is known about such spontaneously occurring plasticity. With multineuron patch-clamp recordings, Le Bé and Markram [12] recently demonstrated that synaptic connectivity between pyramidal cells in rat neocortical slices displays spontaneous rewiring during a period of hours. There is also morphological evidence that hippocampal slices undergo a spontaneous and rapid increase in synapses within 2 hr after slicing [13]. But only few studies have addressed the functional linkage between the ‘pattern’ of spontaneous activity and the resultant plasticity [14]–[17].

Cortical neurons display various patterns of spontaneous activity, depending on the behavioral state of the animal [18], [19]. Neuronal activity during slow-wave sleep and behavior immobility is characterized by slow-wave oscillations and occasional spindle waves in encephalograms, whereas active brain states, including waking and sleep with rapid eye movement (REM), are dominated by faster oscillations in the beta and gamma frequency ranges [18]. It remains controversial whether hippocampal neurons exhibit slow-wave oscillations with bistable membrane potentials [20], [21]. Hahn et al. [22], [23] observed slow oscillation-associated firing modulations in both hippocampal pyramidal cells and interneurons in urethane-anesthetized mice, whereas Isomura et al. [24] failed to find slow oscillations in hippocampal neurons of urethane-anesthetized rats. In this study, we provide evidence that in slice preparations, hippocampal CA3 pyramidal cells exhibit slow oscillations of membrane potential.

The hippocampal CA3 area contains a multiplex network that receives local synaptic inputs and two cortical inputs [25]. The temporoammonic/perforant pathway is a direct input from the entorhinal cortex and is thought to convey spatial information [26]. The mossy fiber pathway mediates an indirect input from the entorhinal cortex. Moreover, CA3 pyramidal neurons synapse with other CA3 pyramidal neurons through the associational/commissural pathway, providing an internal source of excitatory inputs. This “recurrent network” architecture is theoretically considered to yield attractor dynamics underlying associative memory [27]. Here we report that the efficacy of these synapses is modulated by spontaneous slow oscillations of postsynaptic CA3 pyramidal cells with different learning rules and that the synaptic plasticity is heterogenous, complex, and time-varying. We seek to experimentally identify the mechanism and rule of this plasticity and numerically address the functional significance of this plasticity.

## Results

### UP states of hippocampal neurons

**Figure 1:**
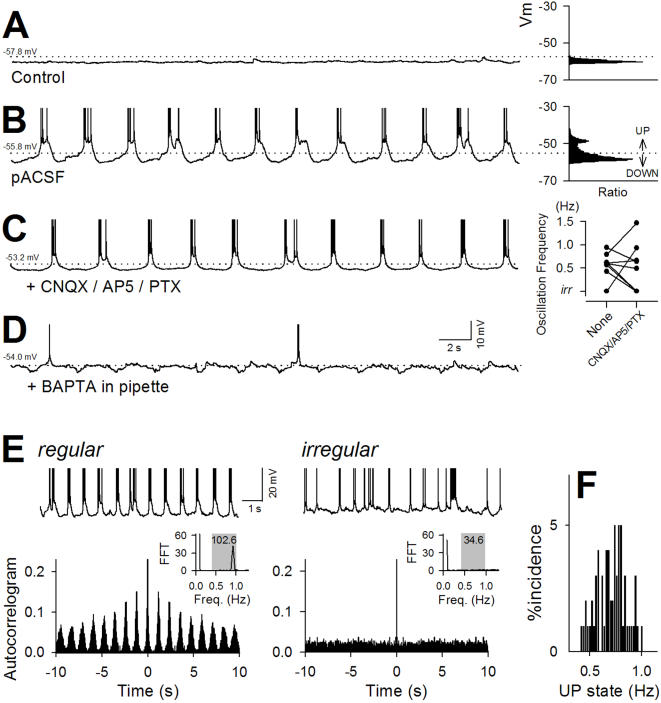
CA3 pyramidal cells show slow oscillations with UP states under *in vivo*-like ionic circumstances. *A–D*, Representative intracellular traces recorded in control ACSF (A) and pACSF in the absence (B) or presence of a combination of CNQX, AP5, and picrotoxin (PTX) (C) or BAPTA (D). CNQX/AP5/PTX were bath-applied, and BAPTA was intracellularly loaded through patch pipettes. After exposure to pACSF, CA3 pyramidal neurons, which remained at resting membrane potentials in control ACSF (A), displayed slow UP-DOWN oscillations (B). Dotted lines indicate the membrane potentials at the 0.1% significance level estimated from the Gaussian curve fit to the DOWN membrane potentials (A: −57.8, B: −55.8, C: −53.2, and D: 54.0 mV), above which level membrane potential was regarded as UP states. The oscillations maintained even in the presence of CNQX/AP5/picrotoxin (PTX), but the oscillation frequencies were modulated. In the right-hand plot of the panel C, the UP-DOWN frequencies were compared in identical cells (connected line) before (None) and after application of CNQX/AP5/PTX. *E*, Regular and irregular oscillations induced by pACSF. Autocorrelograms (bottom) were constructed from intracellular traces (top) to calculate the Fourier transformational integral in the 0.4 to 1.0-Hz range (bottom inset). If this value was higher than 50, the oscillation was regarded as a regular oscillation (left), otherwise as an irregular oscillation (right), because this regularity criteria seems suitable to eye inspection. *F*, Distribution of the mean frequency of regular UP-DOWN oscillations (n = 61).

CA3 pyramidal neurons were visually identified in hippocampal slices prepared from postnatal 14 to 17-d-old rats, and whole-cell patch-clamp recordings were made in current-clamp mode. In standard (*i.e.*, widely used) artificial cerebrospinal fluid (ACSF), the resting membrane potentials were stable, and few action potentials occurred (Figure 1A). When ACSF was modified to mimic the extracellular ionic composition *in vivo* (physiologic ACSF; pASCF) [28]–[36], neurons started to display slow (<1 Hz) fluctuations of subthreshold membrane potentials with action potential trains (Figure 1B). Membrane potentials had bistable voltage distributions (Figure 1B right), typified by the so-called UP and DOWN states [37]–[39]. In more than 70% of the neurons, the oscillation rhythms were regular, and the frequencies were, on average, 0.7±0.2 Hz, ranging from 0.4 to 1.0 Hz (Figure 1E&F), whereas the remaining neurons displayed no apparent periodicity, referred to here as irregular oscillations (Figure 1E) (for definition, see the legend of Figure 1). Incidentally, using a hippocampal slice prepared from a human patient with temporal lobe epilepsy (Figure S1), we found that a dentate granule cell also showed similar UP and DOWN alternations. We could not find healthy pyramidal cells because of severe hippocampal sclerosis, but these results suggest that the capability of showing UP and DOWN states is prevailing among hippocampal neuron types as well as species.

Even though slices were bathed in an inhibitor cocktail consisting of the non-NMDA receptor antagonist 6-cyano-7-nitroquinoxaline-2,3-dione (CNQX, 20 µM), the NMDA receptor antagonist d,l-2-Amino-5-phosphonopentanoic acid (d,l-AP5, 50 µM), and the GABA_A_ receptor antagonist picrotoxin (50 µM), slow oscillations were sustained in 6 of 9 neurons, (Figure 1C), although their frequencies were often altered (Figure 1C right). In three other neurons, the oscillations were shifted to irregular fluctuations, but spontaneous activity *per se* was preserved (Figure 1C right). Therefore, ionotropic synaptic transmission is not essential for the generation of oscillatory activity but coordinates the oscillation properties. The oscillations were markedly attenuated when patch pipettes were loaded with 10 mM BAPTA, a Ca^2+^ chelator, and hence, they were dependent on intracellular Ca^2+^ dynamics in the recorded neurons (Figure 1D, n = 4).

### Spontaneous activity-induced synaptic plasticity

**Figure 2:**
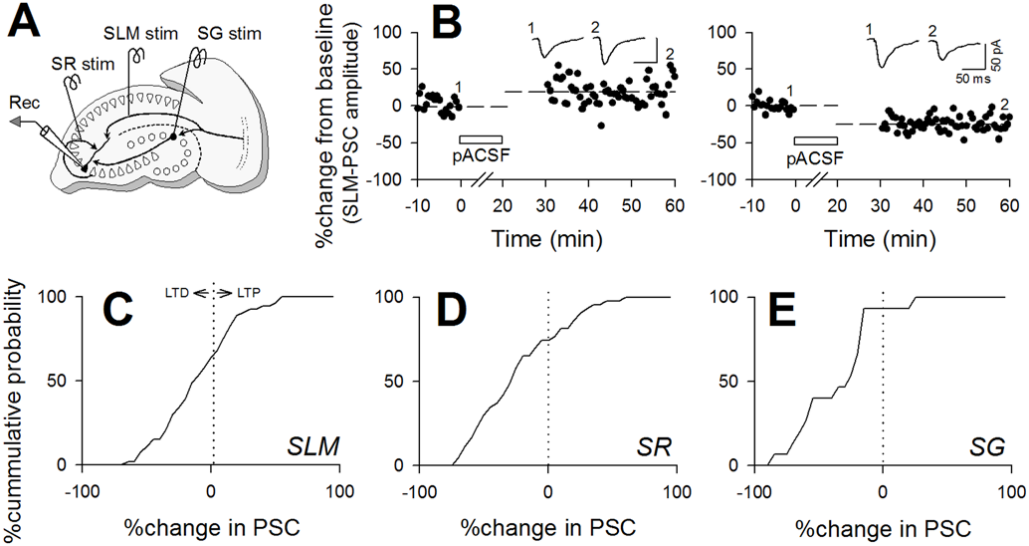
Spontaneous slow oscillations induce bidirectional long-term plasticity at three types of hippocampal CA3 synapses. *A*, Schematic illustration of experimental procedures for recording synaptic responses of CA3 pyramidal cells. Three stimulating electrodes were placed on stratum lacunosum-moleculare (SLM), radiatum (SR), and granulosum (SG) to activate temporoammonic, associational/commissural, and mossy fiber pathways, respectively. *B*, Examples of the time courses in PSCs evoked by SLM stimuli (SLM-PSC) before and after a 20-min exposure to pACSF. pACSF induced a 16.9% increase (left) or a 32.7% decrease in PSC amplitudes (right). *C–E,* Cumulative probabilities of pACSF-induced persistent changes in PSCs evoked by stimulation of SLM (C), SR (D), and SG (E). The zero intercepts were 64.1% (n = 63), 74.4% (n = 59), and 94.4% (n = 18) for SLM-evoked, SR-evoked, and SG-evoked PSCs, respectively.

**Figure 3:**
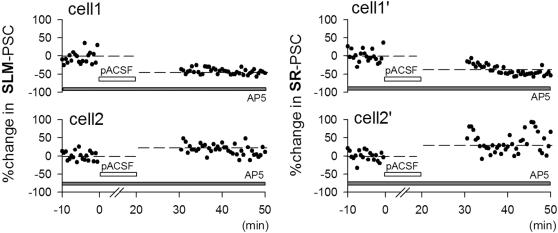
pACSF-induced bidirectional synaptic modification does not require NMDA receptor activity. Even when slices were continuously perfused with 50 µM d,l-AP5 to block NMDA receptors, synaptic modification occurred after pACSF application. The same results were observed in all 7 cases tested.

We examined how these spontaneous neuronal activities modify synaptic connectivity. Test stimuli were applied to the stratum lacunosum-moleculare (SLM), stratum radiatum (SR), and stratum granulosum (SG) to activate temporoammonic, associational/commissural, and mossy fiber pathways, respectively. Evoked excitatory postsynaptic currents (PSC) were recorded from voltage-clamped CA3 pyramidal cells (Figure 2A). After monitoring basal responses, we stopped delivering test stimuli and perfused slices with pACSF for 20 min in current-clamp mode to provoke spontaneous activity. Ten minutes after washing out pACSF, test stimulation was restarted in voltage-clamp mode, and PSC amplitude was compared to the pre-pACSF baseline level. In most cases, the PSC amplitude was altered. The direction and magnitude of this synaptic modification varied from case to case. Two examples of SLM stimulation-evoked responses are shown in Figure 2B; one cell showed long-term potentiation-like phenomenon (LTP, left), whereas the other showed long-term depression-like phenomenon (LTD, right). All data are summarized for SLM-evoked (Figure 2C), SR-evoked (Figure 2D), and SG-evoked PSCs (Figure 2E). After exposure to pACSF, SLM-evoked and SR-evoked PSCs were depressed in 64.1% neurons (n = 63) and 74.4% neurons (n = 59), respectively, whereas MF-evoked PSCs were depressed in almost all cases (17 of 18 neurons). In some slices, we monitored the synaptic modification for up to 75 min after pACSF washout, but we did not find that the altered PSCs spontaneously recovered to the pre-pACSF level. Thus, the plasticity is robust and long-lasting. Because pACSF-induced synaptic modification occurred even in the presence of 50 µM d,l-AP5 (Figure 3). Thus, the plasticity does not require NMDA receptor activity.

**Figure 4:**
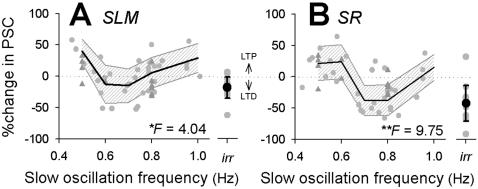
Frequencies of slow oscillations correlate with the direction and magnitude of synaptic plasticity. Relationships between oscillation frequencies during pACSF application and pACSF-induced changes in PSCs evoked by SLM (A, n = 49) and SR stimulation (B, n = 45). Each dot represents a single cell. Means±SDs are indicated by thick lines and shaded areas (0.1-Hz step). One-way ANOVA indicated significant correlations with slow-oscillation frequency: *F_4,28_* = 4.04, *P* = 0.010 (SLM); *F_4,28_* = 9.75, *P* = 0.00007 (SR). Irregular oscillations (*irr*) tended to induce LTD at both types of synapses (n = each 14). For other parameters, see Figure S1. Triangle symbols are experiments with current injection (see Figure 9).

Various parameters of slow oscillations were computed to determine which single parameter gives the best prediction for the direction and magnitude of synaptic plasticity (Figure 4&Figure S2). One-way analysis of variance (ANOVA) revealed that among ten parameters tested here, the mean frequency of slow-wave oscillations, which was calculated by the averaged time interval between two successive UP states, showed the highest correlation, that is, both SLM (Figure 4A) and SR (Figure 4B) plasticity were weakly, but significantly, correlated with the mean frequency of slow oscillations (SLM, *F_4,28_* = 4.04, *P* = 0.010; SR, *F_4,28_* = 9.75, *P* = 0.00007). In both synaptic pathways, the relationships were roughly U-shaped. LTD tended to occur after slow oscillations in the middle-frequency range, and LTP was dominant at higher and lower frequencies (Figure 4). This frequency preference, however, seemed to differ between SLM and SR; SLM favored LTD in the 0.6 to 0.7-Hz range, whereas SR favored LTD in the 0.7 to 0.8-Hz range. Irregular oscillations tended to induce LTD in both SLM and SR pathways (Figure 4).

**Figure 5:**
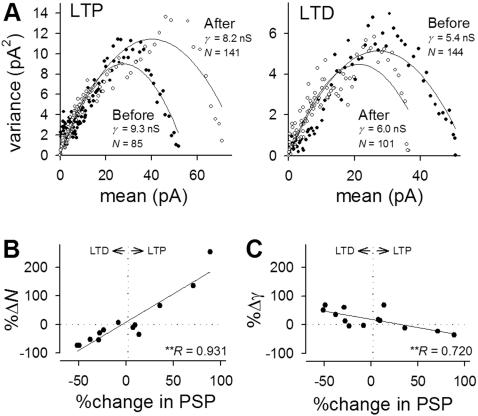
Non-stationary fluctuation analysis on pACSF-induced persistent changes in SLM-evoked PSCs. *A–B*. Representative distributions of current variance as a function of the level of scaled ensemble average current for the PSCs before (closed circles) and after (open circles) LTP (A) and LTD induction (B). *C*, Summarized data of changes in the estimated number of synaptic receptor channels following pACSF application. Changes in PSCs were positively correlated with changes in numbers. *D*, Summarized data of changes in estimated single channel conductance following pACSF application. Changes in PSCs were negatively correlated with changes in channel conductance. **P<0.01, Pearson's test.

Non-stationary fluctuation analysis was applied to assess the expression mechanisms of pACSF-induced synaptic modification. Figure 5A indicates representative plots of current variance as a function of scaled ensemble average current in PSCs before and after LTP or LTD. Data are summarized in Figure 5B and C (n = 13). pACSF-induced changes in PSCs showed a positive correlation with those in estimated number of synaptic receptor channels (Figure 5B) and a slightly, but significantly, negative correlation with those in estimated single channel conductance (Figure 5C). These results will be discussed below

### Spatiotemporal diversity of slow oscillations and synaptic plasticity

**Figure 6:**
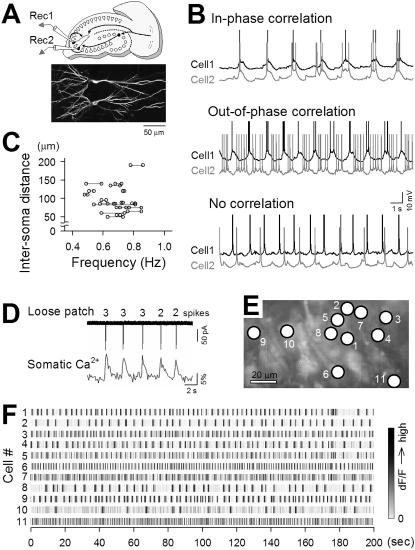
CA3 networks display diverse, mostly uncorrelated, slow oscillations. *A*, Schematic drawing of dual patch-clamp recordings (top) and confocal image of two CA3 pyramidal cells labeled with biotin-streptavidin Alexa-488 (bottom). *B*, Representative intracellular traces simultaneously recorded from two pyramidal cells (cell1 and cell2) in pACSF. In some pairs, slow oscillations were synchronized in-phase (top) or out-of-phase (middle), but many pairs (78.8%) did not show apparent synchronization (bottom). *C*, Distance between two recorded cells versus their slow-wave frequencies. There was no relationship between cell location and synchronization (n = 25 pairs). *D,* Simultaneous loose-patch-clamp recordings and confocal monitoring of neurons bulk-loaded with Oregon green 488 BAPTA-1 revealed that somatic Ca^2+^ transients reflect action potentials. Numbers above the trace indicate the numbers of spikes involved in the corresponding burst trains. *E–F*, Fast confocal scanning of Ca^2+^ transients of 11 neurons, the locations of which are shown in panel E, revealed that different cells generated Ca^2+^ transients at different frequencies. Fluorescent intensities were normalized in each cell, indicated in gray scale.

Slow oscillations varied in frequency from slice to slice and were refractory to pharmacologic blockade of synaptic transmission. These observations led us to examine whether the oscillations were diverse among cells in a slice. We performed patch-clamp recordings simultaneously from two CA3 pyramidal cells in pACSF (Figure 6A). A few cell pairs generated synchronized UP states in phase (n = 5) or out-of phase (n = 2), but about 80% of the cell pairs (n = 26) did not show apparent correlations; they generated slow oscillations at different frequencies (Figure 6B). The spatial distance between the somata of cell pairs was plotted versus the oscillation frequencies of these cells (Figure 6C), but no relationship was found between them, that is, more closely located pairs did not necessarily show higher correlations.

To further confirm the oscillation diversity, hippocampal slices were bolus-loaded with Oregon green 488 BAPTA-1AM and imaged with a high speed-scanning confocal microscope (Movie S1) [40]. Loose-patch recordings revealed that somatic Ca^2+^ transients reliably reflected action potential trains and thus can be used as a reporter of UP state timings (Figure 6D). Fluorescence signals of 11 neurons in a slice exposed to pACSF are shown in Figure 6E and F. Of these 11 neurons, 6 neurons generated regular slow oscillations, the frequencies and phases of which were different between the cells. Similar results were obtained in all 8 slices tested.

**Figure 7:**
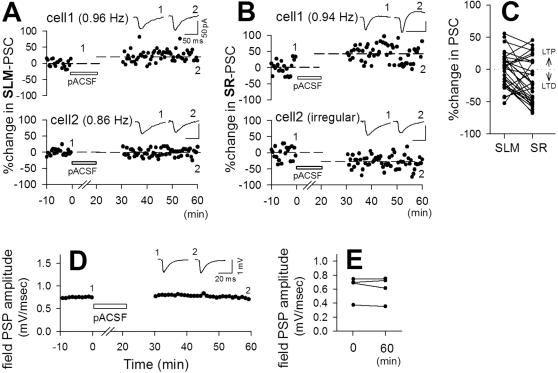
Diversity in pACSF-induced synaptic modification in a network. *A–B*, Time course of SLM-evoked (A) and SR-evoked PSCs (B) in simultaneously recorded cell pairs. Different cells showed different levels of synaptic plasticity in the same slices. *A*, Cell1 showed 0.96-Hz oscillations and a 23.4% potentiation of PSCs, whereas cell2 showed 0.86-Hz oscillations and no plasticity (−0.6%). *B*, Cell1 showed 0.94-Hz oscillations and a 48.6% potentiation, whereas cell2 showed irregular oscillations and a 28.4% depression. *C*, pACSF-induced changes in SLM-evoked and SR-evoked PSCs recorded simultaneously from single cells (n = 31). *D–E*, No plastic change was observed when synaptic transmission was monitored by field recording. *D*, Representative time course of SR-evoked field postsynaptic potentials (PSPs). No stimuli were delivered from 0 to 30 min. *E*, Summary of changes in SR-evoked field PSPs before and after pACSF application (n = 4 slices). Field recordings failed to find pACSF-induced synaptic plasticity.

As we observed above, slow oscillations were diverse among neurons, On the other hand, oscillation frequencies determine the level of synaptic plasticity. We thus expected that oscillation-induced synaptic plasticity was also diverse in single slices. This was indeed the case. Typical data of dual patch-clamp recordings are shown in Figure 7A (SLM-evoked PSCs) and Figure 7B (SR-evoked PSCs). In pACSF, different neurons generated different oscillation frequencies (or irregular oscillations), and thereafter, they showed different levels of synaptic plasticity (SLM, n = 21 pairs; SR, n = 22 pairs); LTP and LTD were sometimes simultaneously expressed at the same synaptic pathway onto different cells (e.g., Figure 7B). We next used two stimulating electrodes to compare SLM and SR synaptic plasticity within single cells (Figure 7C, n = 31 cells). In some cases, LTP and LTD were coexpressed at different synaptic pathways onto the identical cells, but in many cells, the direction of synaptic plasticity was the same between SLM and SR (paired-*t* test, *t*_56_ = 2.60, *p* = 0.01), probably because the plasticity induction rule was similar between SLM and SR (see Figure 4). Importantly, pACSF-induced plasticity was not observed when we monitored ‘field’ postsynaptic potentials, which reflected mixed responses of many synapses surrounding the tip of the recording electrode (Figure 7D&E). The total amount of LTP and LTD may be balanced in space and hence averaged out in the bulk measure ‘field PSP’.

**Figure 8:**
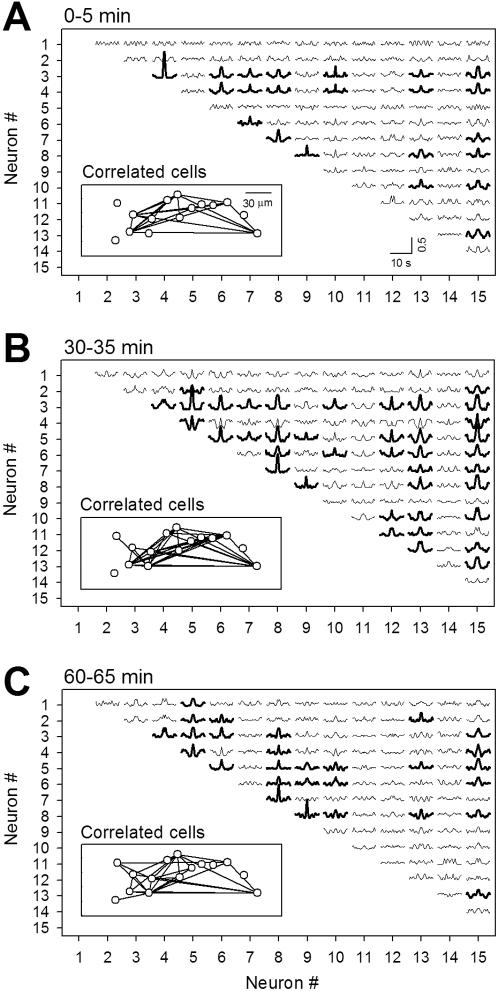
Network structures of slow oscillations change over time during prolonged pACSF application. Crosscorrelogram of somatic Ca^2+^ fluorescence signals of 15 CA3 neurons in a hippocampal slice. pACSF were continuously bath-applied, and each 5-min segment of the fluorescence traces was used to calculate crosscorrelogram (A: 0–5 min; B: 30–35 min; C: 60–65 min). Neuron pairs that showed correlations with the peak amplitude of more than 0.5 were drawn with thick lines in the correlogram panels and topologically linked in the physical cell maps (insets). Note the combination of correlated cell pairs changed over time.

**Figure 9:**
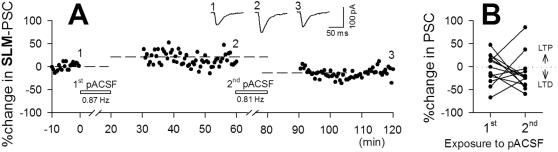
Different levels of synaptic plasticity are induced by repeated exposure to pACSF. *A*, Representative data of SLM-evoked PSCs. The first pACSF application induced 0.87-Hz oscillations and a 16.4% potentiation, and 60 min later, the second pACSF induced 0.81-Hz oscillations and a 23.9% depression. Data are summarized in panel B (n = 12).

The diversity of synaptic plasticity indicates that global, complex remodeling of network connectivity occurs during spontaneous oscillations. Because network activity and the strength of individual synapses are reciprocally linked [14], the patterns of spontaneous activity, which arise partly from networks, could vary over time via ongoing plasticity. Indeed, multineuron calcium imaging revealed that the oscillation rhythms of individual neurons and the sets of correlated neuron pairs were changed during a long application of pACSF (Figure 8). This implies that the same neurons exhibit different levels of synaptic plasticity at different times. To address this possibility, we applied 20-min applications of pACSF twice, separated by an interval of 40 min. The cell shown in Figure 9A displayed slow oscillations at 0.87 and 0.81 Hz during the first and second pACSF challenges, respectively, and expressed LTP and then LTD, respectively. Data are summarized in Figure 9B (n = 12 cells). There was no apparent relationship between the levels of synaptic plasticity induced by the first and second pACSF challenges (paired-*t* test, *t*_24_ = 0.75, *p* = 0.46). Thus, the network reorganization is not only spatially heterogenous, but is also temporally diverse.

### Postsynaptic induction of oscillation-induced synaptic plasticity

**Figure 10:**
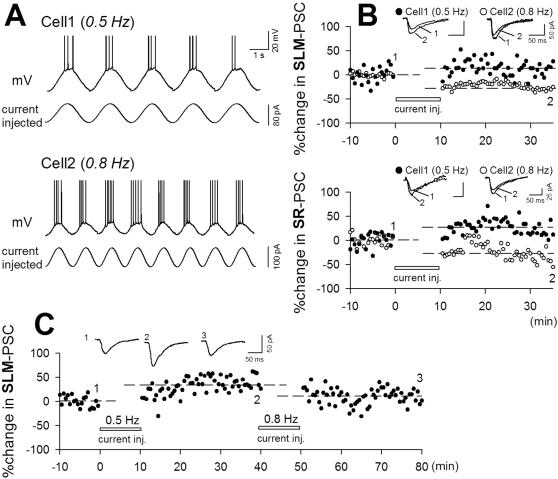
Forced slow oscillations of postsynaptic membrane potentials induce bidirectional synaptic modifications. *A*, Sinusoidal currents with different frequencies (cell1, 0.5 Hz; cell2, 0.8 Hz) were simultaneously injected into two neurons. Both neurons showed oscillation-locked spiking activity. *B*, Time course of SLM-evoked (top) or SR-evoked PSCs (bottom) in cell1 (closed circles, 0.5 Hz) and cell2 (open circles, 0.8 Hz) in a single slice following the current injection. Top: 13.0% potentiation in cell1 and 38.3% depression in cell2. Bottom: 25.9% potentiation in cell1 and 29.4% depression in cell2. Data for other experiments are shown as triangle symbols in figure 4 (SLM, n = 12; SR, n = 13). *C*, A neuron displayed LTP and then LTD in response to 0.5-Hz and 0.8-Hz current injection, respectively.

Do postsynaptic slow oscillations alone induce synaptic modifications? In control ACSF, sinusoidal currents that mimicked slow oscillations were injected into dual-patched CA3 neurons at different cycle frequencies. For each cell, the sinusoidal amplitude was set to the minimal level that reliably produced a few spikes per cycle (50–120 pA). In the example shown in Figure 10B, cell1 and cell2 were activated by sinusoidal currents with amplitudes of 80 pA and 100 pA, respectively (Figure 10A). When current at different frequencies was injected (0.5 Hz for cell1 and 0.8 Hz for cell2), cell1 and cell2 displayed different levels of synaptic modification of SLM-evoked and SR-evoked PSCs (Figure 10B). The other data are shown as triangle symbols in Figure 4 (n = 25). When sinusoidal current at different frequencies was repeatedly injected into the same cells, they displayed different levels of synaptic plasticity (Figure 10C). These results indicate that postsynaptic oscillations are sufficient to induce synaptic plasticity.

**Figure 11:**
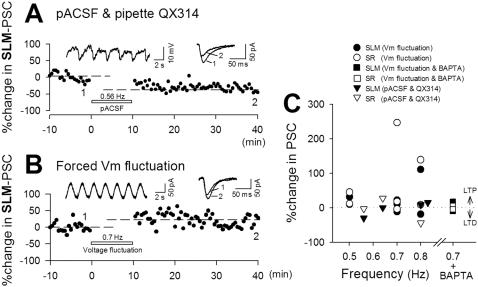
Subthreshold slow oscillations induce bidirectional synaptic modifications. *A*, Responses were recorded with a patch pipette loaded with 5 µM QX314. pACSF elicited slow oscillations without action potentials and still induced synaptic plasticity. Data are summarized in panel *C* (closed triangles: SLM, n = 4, open triangles: SR, n = 3). *B*, A neuron was held in voltage-clamp configuration, and its membrane potential was forced to oscillate via voltage commands (from −70 to −45 mV at 0.7 Hz). No action potential occurred, but LTP took place. Data are summarized in the panel *C* (closed circles: SLM, n = 7; open circles: SR, n = 6). The same experiments were repeated with a pipette loaded with 10 mM BAPTA (closed squares: SLM, n = 4; open squares: SR, n = 3).

To examine whether spikes during oscillations are required for synaptic plasticity, we applied sinusoidal fluctuations of subthreshold membrane potential through voltage commands ranging from −70 to −45 mV in voltage-clamped cells. These forced oscillations did not evoke spikes but still induced synaptic plasticity (Figure 11B). As another test, patch pipettes were loaded with 5 µM QX-314, a voltage-sensitive Na^+^ channel blocker, to prevent spike generation and slices were perfused with pACSF. Neurons displayed UP-DOWN oscillations without spikes (Figure 11A, inset) and still expressed synaptic plasticity. Data were plotted in Figure 11C (triangles). We thus conclude that the synaptic plasticity was induced even without postsynaptic spikes. But without spikes, it did not any longer conform to the induction rule seen in pACSF-induced plasticity, that is, there was no correlation between UP-DOWN frequencies and the levels of resultant synaptic plasticity (Figure 11C versus Figure 4). It is likely, therefore, that although synaptic plasticity occurs through subthreshold oscillations alone, the plasticity rule is shaped by postsynaptic spikes.

We sought to examine whether postsynaptic Ca^2+^ is required to induce synaptic plasticity. Unfortunately, the effect of intracellular Ca^2+^ chelating on pACSF-induced plasticity could not be addressed, because pACSF-induced slow oscillations *per se* were sensitive to Ca^2+^ chelatoring (Figure 1D). Instead, neurons were voltage-clamped, and 0.7-Hz membrane potential oscillations were produced by voltage commands through pipettes loaded with 10 mM BAPTA. Under these conditions, no synaptic plasticity occurred (Figure 11C squares). Thus, voltage oscillation-induced synaptic plasticity is likely to be Ca^2+^-dependend.

### Functional significance of slow oscillation-induced plasticity

**Figure 12:**
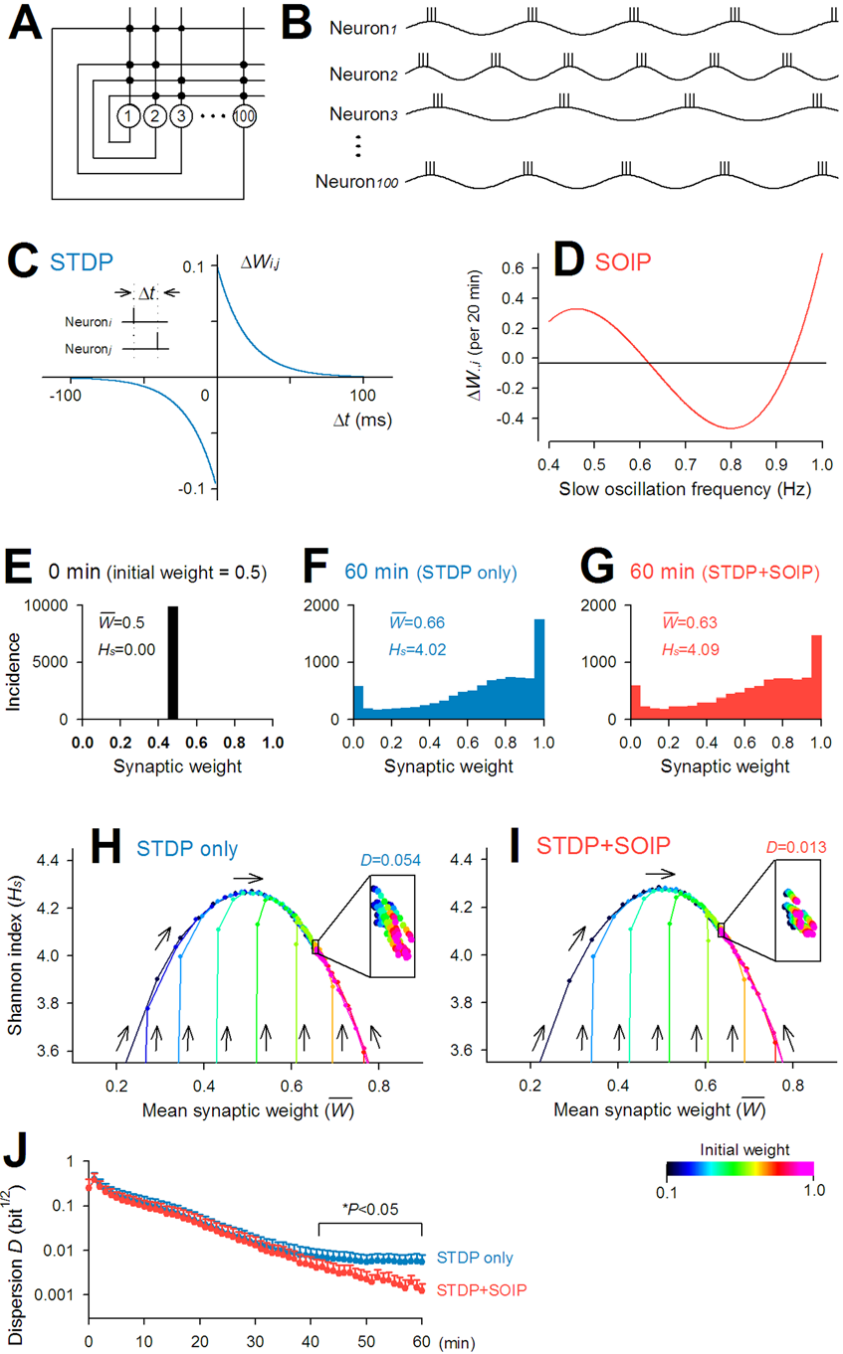
Slow oscillation-induced plasticity (SOIP) enhances the stability of network state: a numerical simulation study. *A*, A hundred neurons are connected in an all-to-all fashion except themselves. *B*, Membrane potentials of these neurons are oscillated at different slow oscillation rhythms, in every cycle of which they fire 3 spikes at 40 Hz. *C,* The rule of spike-timing dependent plasticity (STDP). *D* *,* The rule of slow oscillation-induced plasticity (SOIP). *E* *–* *G*, Example of simulation. Synaptic weights are set to 0.5 (*E*) and updated along slow oscillations for 60 min with STDP only (*F*) or with a combination of STDP and SOIP (*G*). The distribution of synaptic weights is evaluated by the mean weight 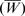 and Shannon index (*H_s_*). *H,I,* Time course of the change in the space of 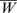 versus *H_s_* during a updating period of 60 min with STDP only (*H*) and with a combination of STDP and SOIP (*I*). The weights are initially uniform, being set to a value ranging from 0.1 to 1.0. The insets show the distribution for the last 10 min. The state of a network with STDP alone is more unstable. The instability (*i.e.*, data point dispersion) is captured by .the sum of the Euclidian distance from the gravity (*D*). *J,* Time course of the dispersion *D* of network states with STDP only (blue) and with both STDP and SOIP (red). Statistical significance was determined with Welch's test.

To conjecture the function of pACSF-induced plasticity, we compared network dynamics *in silico* between the cases with and without slow oscillation-induced plasticity (SOIP). We constructed a recurrent network, in which 100 neurons were synaptically connected in an all-to-all fashion (Figure 12A). Every neuron continued to show regular slow oscillations (Figure 12B), the frequency of which was randomly assigned so as to conform a pseudo-Gaussian distribution of 0.7±0.2 Hz, ranging from 0.4 to 1.0 Hz (see Figure 1F). At a phase of 90° in each oscillation cycle, neurons fired three action potentials (see Figure S2A_7_) (Figure 12B). These action potentials were separated with at an interval of 25 ms (40 Hz = gamma frequency). Synaptic weights took values ranging from 0 to 1 and were initially identical at all synapses. The neural network self-organized these synaptic weights, according to the following two rules: i) Spike-timing dependent plasticity (STDP) (Figure 12C). The update rule was extracted from the electrophysiological data reported by Bi and Poo [11]. If neuron*_i_* fired before neuron*_j_* with a time difference (Δ*t*) being 0 ms and more (Δ*t*≥0), the change in the synaptic weight from neuron*_i_* to neuron*_j_* was given as 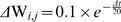, while for Δ*t* being less than 0 (Δ*t*<0), 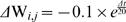. ii) SOIP (Figure 12D). The update rule was obtained by fitting the Figure 4B data to a cubic equation with a least square method. For 20 min, 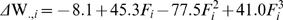, where *F_i_* was the slow oscillation frequency of neuron*_i_* (in Hz). Note that according to this equation, all synapses projecting to neuron*_i_* was uniformly strengthened or weakened. This updating process was applied every minute.

An example of simulation was demonstrated in Figure 12E–G, which show the distributions of synaptic weight before and after a 60-min period of synaptic update with and without SOIP. The distribution was evaluated with two parameters, *i,e.*, i) the mean synaptic weight 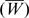 and ii) Shannon index (*H_s_*), a diversity measure. The Shannon index was defined as 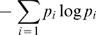, where *p_i_* was the fraction of synaptic weight belonging to the *i*-th bin. The bin width was 0.05. In this example, the initial synaptic weights were 0.5, Thus, 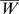 and *H_s_* were 0.5 and 0, respectively (Figure 12E). Figure 12F and G indicate the distributions 60 min after simulation with STDP alone and a combination of STDP and SOIP, respectively. The final 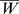 and *H_s_* were very similar between these cases, suggesting that the state converged on a similar configuration of synaptic matrix, irrespective of whether or not SOIP was incorporated into the network. Figure 12H and I indicate the state trajectories of the networks in the space of 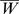 versus *H_s_* for 60 min. The initial synaptic weights were set to 0.1, 0.2, 0.3, 0.4, 0.5, 0.6, 0.7, 0.8, 0.9, or 1.0, and the neurons were given the same set of slow oscillation rhythms across simulations. Irrespective of the initial synaptic weights, the network state was eventually attracted to the same narrow space. Thus, the stable network state was insensitive to the initial synaptic conditions. But in the vicinity of the stable point, the network state was jittered, and the network with STDP alone seemed to be more unstable than that with both STDP and SOIP (Figure 12H and I, insets). We defined the state dispersion (i.e., network instability) as the sum of the Euclidian distances from individual data points to the gravity of all points (*D*). As the network state came close to the stable point, the *D* value of the network with STDP only was significantly higher than that with both STDP and SOIP. Thus, the network embedded with SOIP is more stable.

## Discussion

### Intrinsic nature of slow oscillations

Neuronal oscillations emerge with various rhythm frequencies, depending on animal's behavioral state [18], [19]. Electroencephalographic slow-wave oscillations are usually observed during non-REM sleep and associated with UP-DOWN alternations in membrane potential of cortical and subcortical neurons [35], [37], [38], [39], [41]. The slow-wave state, often accompanied by hippocampal sharp wave/ripples, is thought to contribute to memory consolidation [42], yet it remains controversial whether hippocampal neurons display intracellular slow oscillations [22], [23], [24]. In the present study, we demonstrated that CA3 pyramidal cells, even if synaptically isolated, produce slow oscillations with UP-DOWN bistability. We do not know the reason why Isomura et al. [24] failed to find similar UP-DOWN oscillations. One possibility may be the difference in preparations. Isomura et al. [24] used urethane-anesthetized rats, whereas we used slice preparations of the rodent and human. But Hahn et al. [22], [23] used urethane-anesthetized mice and observed UP-DOWN-locked spike modulations in CA1 and CA3 pyramidal cells, dentate granule cells as well as interneurons. Moreover, Zhang et al. [43] reported slow oscillations in membrane potentials of hippocampal neurons treated with Cs^+^. We therefore conclude, at least, that hippocampal neurons possess the ability to generate UP-DOWN membrane bistability, although the physiological relevance remains unclear.

In general, rhythmic fluctuations of neuronal activity arise from intrinsic and/or network properties [19], [44]. As for intrinsic oscillations, a key is ion channel activity, *e.g.*, persistent sodium current [45], [46] and hyperpolarization-activated current [47], [48], whereas the network sources are mainly given in the form of synchronous synaptic inputs [35], [49], [50]. In our preparations, slow oscillations were altered by a blockade of synaptic transmission, but the bistable oscillations *per se* were preserved. This is unique, different from both the neocortex and cerebellum. Neocortical UP states are network-driven and require recurrent synaptic barrages with balanced excitation and inhibition [35], [49], [50], whereas UP states of entorhinal layer 5 neurons [51] and cerebellar Purkinje cells [52] are purely intrinsic, representing single-cell attractors. Our observation shows that hippocampal neurons can show UP-DOWN alternations independently of the network, like a pace maker or central pattern generator [44], [53] and that the activity pattern and periodicity are subject to network activity. These neurons might intrinsically generate weak rhythmic activity that can be entrained or resonated to network activity, thereby generating a rich variety of UP-DOWN frequencies.

Hippocampal pyramidal cells are heterogenous. Single-cell microarray analysis reveals that the gene expression profile differs among hippocampal pyramidal cells [54]. In behaving animals, different hippocampal neurons have different “place fields”; and they fire selectively in different regions of an environment [55]. A subset of hippocampal neurons tends to fire in synchrony with theta-wave oscillations, but others do not [56]–[58]. We found that UP-DOWN oscillations of CA3 pyramidal neurons were also diverse and even time-varying. This is consistent, in part, with a report by Hahn et al. [23] that demonstrates the diversity of UP-DOWN modulations of CA3 neurons in anaesthetized mice. Given that in the neocortex, UP states are mostly synchronized between neurons in a local network [35], [49], [59], [60], [61], the activity we describe here represents a unique event of spontaneous activity.

### Diversity and mechanisms of synaptic plasticity

Corresponding to the heterogeneity of hippocampal slow oscillations, the resulting plasticity was also diverse and complex; LTP and LTD were co-expressed in the same network and even the same neurons. In general, application of artificial electrical stimulation, *e.g.*, tetanic stimulation, theta burst stimulation, or low-frequency stimulation, induces a homogenous change in synaptic efficacy [4], [5], [7]. Chemically induced neuronal activity also results in monotonous synaptic plasticity [62]–[64]. Compared with artificially induced synaptic plasticity, naturally occurring synaptic modification, as observed here, is much more complex and diverse (see also [12]).

pACSF-induced plasticity depended on postsynaptic membrane oscillations and postsynaptic Ca^2+^ dynamics, but it did not require NMDA receptor activity. The mechanism is, therefore, different from “conventional” NMDA receptor-dependent synaptic plasticity [1]. Rather, it is similar to that of spontaneous rewiring of synaptic connectivity in neocortical slices, which is also NMDA receptor-independent [12]. Unfortunately, we could not address the mechanisms in detail, because in our experiments, spontaneous slow oscillations *per se* were used as a conditioning stimulus to induce synaptic plasticity. Pharmacologic agents and physiologic manipulations that were found to prevent or modulate synaptic plasticity often affected slow oscillations themselves as well, and it is unclear whether the effects were due to direct actions on the fundamental mechanisms underlying synaptic plasticity or simply due to indirect effects via the altered slow oscillations. Instead, we tried to extract a rule that governs the level of synaptic plasticity and found that the UP-DOWN rhythm roughly, though not perfectly, predicts the direction and degree of synaptic plasticity. Importantly, the sinusoidal current injection also induced frequency-dependent plasticity, the direction of which conformed to the rule of pACSF-induced synaptic modification. These observations support the idea that our LTP and LTD were a result of slow oscillations, rather than a random phenomenon.

The present study did not determine the location of expression of pACSF-induced synaptic plasticity, but we hypothesize that the plasticity is postsynaptically expressed, based on the following two reasons: i) postsynaptic subthreshold membrane fluctuations were sufficient to induce the plasticity, ii) most synapses activated by test stimulation were thought to undergo the same direction of plasticity. Note that in our experimental protocol, numerous presynaptic fibers were activated with field stimulation. If individual synapses independently showed LTP or LTD, we could not have observed apparent plastic changes in bulk. Given that neighboring postsynaptic neurons showed different frequencies of slow oscillations and thereby expressed different levels of plasticity even at the same presynaptic pathway (Figure 7A&B), it is plausible that the expression site of the plasticity is primarily postsynaptic. Under this assumption, our non-stationary fluctuation analysis suggests that pACSF-induced plasticity are due to a change in the number of postsynaptic receptor channels. They may be mediated by insertion and removal of glutamate receptors through trafficking and stabilization [65].

### Speculations on physiologic significance of ongoing synaptic plasticity

We demonstrated that hippocampal pyramidal cells update their synaptic weights in response to their own spontaneous activity. Because spontaneous activity is an ongoing endogenous event that is prevailing in the brain *in vivo* and varies from cell to cell [66], [67], the resulting synaptic plasticity continuously occurs in the brain, being diverse at the single-cell (even single-synapse) level. The phenomenon observed here is probably a part of this ongoing huge event. For this reason, the preexisting levels of synaptic plasticity will also be heterogenous at the time when experiments are performed. This may be a reason for the diversity we observed here, and it is also consistent with the findings of Debanne et al. [68] that the margin for potentiation is heterogenous at the single-synapse level, suggesting different levels of already-existing LTP.

As discussed above, pACSF-induced plasticity is a global event that uniformly occurs at virtually all synapses of the oscillating neuron. Intuitively one may deduce that such a global event nonspecifically collapses or erases memory traces in a network, because memory is thought to be stored on a synapse-specific network refinement [1]. From this point of view, intriguing is the hypothesis that activity of hippocampal neurons during slow oscillations contributes to erasing recently learned information in the hippocampus after memory transfer to the neocortex [69]. On the other hand, our simple simulation on a recurrent network has revealed that SOIP enhances the stability of the synaptic matrix. We thus hypothesize that a the diversity of slow oscillations does not help reset memory traces via apparently random synaptic modification but rather makes it possible to etch them more consistently and stably in network architecture.

Reverberation of patterned activity during slow-wave states in non-REM sleep is believed to have a fundamental role in memory retrieval and consolidation [42], [70], [71]. Because cortical connectivity runs down during non-REM sleep [72], slow-wave oscillations represent the brain state devoted to local processing of internal information. This, together with our findings, implies that slow oscillations represent a brain state in which the cortex reorganizes its functional connectivity by referring to internally stored information. Importantly, the pattern of slow oscillations and the content of the resultant plasticity are transmuted over time. In other words, autonomous synaptic modification progressively, almost irreversibly, creates a new cortical network state, and this new state emits a different pattern of spontaneous activity, further creating a new state *ad infinitum* [73]. Such ongoing synaptic plasticity might be linked to a working self-renewal process that embodies functional adaptation to the environment.

## Materials and methods

### Hippocampal slice preparations

Hippocampal slices were prepared from postnatal 14 to 17-d-old Wistar/ST rats (SLC, Shizuoka, Japan), according to the Japanese Pharmacological Society guide for the care and use of laboratory animals. Rats were anesthetized with ether and decapitated. The brains were immersed in ice-cold artificial cerebrospinal fluid (ACSF) consisting of (in mM): 127 NaCl, 26 NaHCO_3_, 1.6 KCl, 1.24 KH_2_PO_4_, 1.3 MgSO_4_, 2.4 CaCl_2_, and 10 glucose, bubbled with 95% O_2_ and 5% CO_2_. Transverse hippocampal slices (400 µm thick) were cut with a ZERO-1 (Dosaka, Osaka, Japan) or a Vibratome 3000 (Vibratome, St. Louis, MO). In some experiments, human brain slices were used under the approval of the ethical committee at Tokyo Women's Medical University (approval number #1147). The preparations were derived from biopsy tissues which had to be removed for the surgical treatment of temporal lobe epilepsy with written informed consent of six patients prior to surgery. Human hippocampal slices were prepared in the same way as used for making rat slices.

### Electrophysiologic recordings

Slices were preincubated for at least 90 min in bubbled ACSF and placed in a recording chamber perfused with ACSF at a rate of 2 to 3 ml/min. Whole-cell patch-clamp recordings were performed with glass pipettes (6–8 MΩ) filled with intracellular solution containing (in mM) 120 K-gluconate, 20 KCl, 10 HEPES, 0.1 CaCl_2_, 4 Mg-ATP, and 0.2 EGTA (pH 7.4, 280–290 mOsm). Recordings were performed with Axopatch 200B and Axopatch 1D amplifiers (Molecular Devices, Union City, CA). CA3 pyramidal cells were identified using an Olympus BX50WI microscope (Tokyo, Japan) and a 40× objective under differential interference contrast control with a C3077-78 CCD camera and a Argus-50 photon-counting image processor (Hamamatsu Photonic Systems, Hamamatsu, Japan). Pipette seal resistances were typically >1 GΩ, and pipette capacitive transients were minimized prior to breakthrough. Post-synaptic currents (PSCs) were studied in voltage-clamp mode at −70 mV holding potential. Baseline PSCs were evoked by 100-µs constant current pulses delivered every 30 s to the SLM, SR, and SG through concentric bipolar microelectrodes with Nihon Kohden SEN3301 stimulators and SS202J current-output isolation units (Tokyo, Japan). When two afferents were activated in identical preparations, they were alternatively stimulated every 15 sec. Stimulation intensity (20–150 µA) was adjusted to evoke a response 50% of the maximal value. Signals were low-pass filtered at 1 kHz, digitized at 10 kHz, and analyzed with pCLAMP 8.0 software (Molecular Devices). To induce spontaneous activity, the whole-cell configuration was switched to current-clamp mode, and ACSF was replaced with physiologic ACSF (pACSF) consisting of (in mM): 127 NaCl, 26 NaHCO_3_, 3.3 KCl, 1.24 KH_2_PO_4_, 1.0 MgSO_4_, 1.0 CaCl_2_, and 10 glucose [28]–[36]. Voltages were not corrected for the theoretical liquid junction potential (approximately 10 mV). Data were discarded if access resistance changed by more than 15% during an experiment. Loose-patch-clamp recordings were performed with glass pipettes filled with ACSF to record single-unit extracellular activity. Field postsynaptic potentials evoked by 50-µs constant current pulses delivered every 30 s were recorded with a glass microelectrode filled with 0.15 M NaCl (∼1 MΩ of resistance). Custom-made Igor software was used for data analysis. Non-stationary fluctuation analysis of synaptic currents was performed as described by Robinson et al. [74]. Briefly, PSCs were aligned by their pre-stimulus baselines. The average response waveform was scaled to the peak of individual responses, and the variance of the fluctuation of the decays around the mean was calculated. This variance was binned, and the single-channel current was then estimated by fitting the data to the following: *σ* ^2^ = *iI*-*I* ^2^/*N*+*b*, where *σ* ^2^ is the variance, *I* is the mean current, *N* is the number of channels activated at the peak, *i* is the single-channel current, and *b* is the background variance, which was subtracted in graph. The single-channel conductance (*γ*) is then *γ* = *i*/*V*, where *V* is the driving force (holding potential-assumed reversal potential of 0 mV). All salts used were obtained from Wako Chemicals (Osaka, Japan). d,l-2-Amino-5-phosphonopentanoic acid (d,l-AP5), 6-cyano-7-nitroquinoxaline-2,3-dione (CNQX), picrotoxin, BAPTA, and QX314 chloride were from Sigma-Aldrich (St. Louis, MO).

### Ca^2+^ imaging

Slices were prepared from postnatal 14 to 17-d-old Wistar/ST rats or postnatal 9 to 12-d-old ICR mice and incubated in a 35-mm dish filled with 2 ml of dye solution for 1 h in a humidified incubator at 37°C in 5% CO_2_. The dye solution contained 10 µl of 0.1% Oregon green 488 BAPTA-1AM/DMSO (Invitrogen, Eugene, OR), 2 µl of 10% Pluronic F-127/DMSO (Invitrogen), 2 µl of 4% sulfinpyrazone/DMSO (Sigma-Aldrich), 2 µl of 5% Cremphor EL/DMSO (Sigma-Aldrich), and 1.6 µl of 1.25% Pronase (Sigma-Aldrich). After washing, slices were incubated at room temperature for at least 30 min, mounted in a recording chamber, and perfused with pACSF. Images (653×492 pixels, 16-bit intensity) were captured at 10–30 Hz with a CSU10 Nipkow-disk confocal unit (Yokogawa, Electric, Tokyo, Japan), cooled CCD cameras (Cascade, Roper Scientific, Tucson, AZ; DV887, Andor, Belfast, Northern Ireland, UK), a AxioSkop2 microscope (Zeiss, Oberkochen, Germany), water-immersion 40× objective (0.8 NA, Achroplan, Zeiss), and Metamorph software (Universal Imaging Corporation, West Chester, PA). Fluorophores were excited at 488-nm line and visualized with a 507-nm long-pass emission filter. Spike times were reconstructed from movies by using custom-written software in NIH ImageJ and Microsoft Visual Basic, as previously described [75]. For each cell, the change in fluorescence, *ΔF*/*F*, was calculated as (F_1_−F_0_)/F_0_, where F_1_ is fluorescence intensity at any time point, and F_0_ is the average baseline intensity.

## Supporting Information

Figure S1 Human hippocampal neurons also display UP-DOWN states. *A*, Biocytin reconstruction of a granule cell recorded in a slice of a hippocampal formation tissue biopsied from a patient with temporal lobe epilepsy. *B*, The neuron shown in the panel *A* was recorded in a current clamp mode. It displayed spontaneous membrane potential bistability with action potentials.
(0.08 MB JPG)

Figure S2 Relationships between synaptic plasticity and various parameters related to membrane oscillations. Changes in SLM-evoked (*A*) and SR-evoked (*B*) PSCs are plotted against (*1*) the regularity of slow oscillation rhythms (coefficient of variation in inter-UP state intervals), (*2*) the average time spent in single UP states, (*3*) the ratio of the total time spent for UP states to the total time recorded, (*4*) the voltage differences between UP and DOWN states, (*5*) the ratio of UP states accompanying spikes to the total UP states, (*6*) the mean firing rate, *i.e.*, spikes per second, during UP states, (*7*) the mean number of spikes per UP state, (*8*) the coefficient of variation (CV) in inter-spike intervals, (*9*) and the total number of spikes during pACSF exposure. For each comparison, one-way ANOVA was performed to determine whether the parameter was correlated with the direction and magnitude of synaptic plasticity, but there was no statistical significance for any parameters tested.
(0.58 MB JPG)

Movie S1 Functional multineuron calcium imaging of UP states of CA3 neurons. The CA3 pyramidal cell layer of a slice bulk-loaded with the Ca2+ indicator Oregon green 488 BAPTA-1 was imaged by a spinning disk confocal microscope at a frame rate of 10 Hz. Different cells displayed Ca2+ transients at different UP-DOWN frequencies.
(1.65 MB MOV)
