# Supplementary figures and images for: Active Hippocampal Networks Undergo Spontaneous Synaptic Modification
Source: PLoS One. 2007 Nov 28;2(11):e1250. doi: 10.1371/journal.pone.0001250 (PMC2082078; doi:10.1371/journal.pone.0001250)

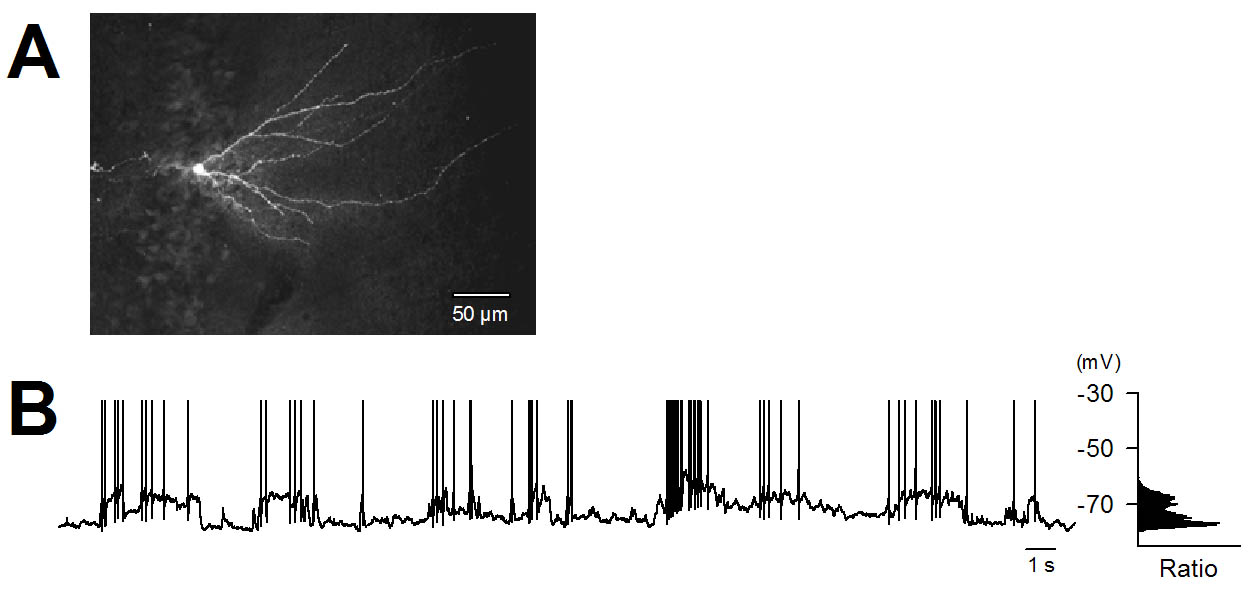

Supplement: Figure S1 — Human hippocampal neurons also display UP-DOWN states. A, Biocytin reconstruction of a granule cell recorded in a slice of a hippocampal formation tissue biopsied from a patient with temporal lobe epilepsy. B, The neuron shown in the panel A was recorded in a current clamp mode. It displayed spontaneous membrane potential bistability with action potentials. (0.08 MB JPG) [file pone.0001250.s001.jpg]

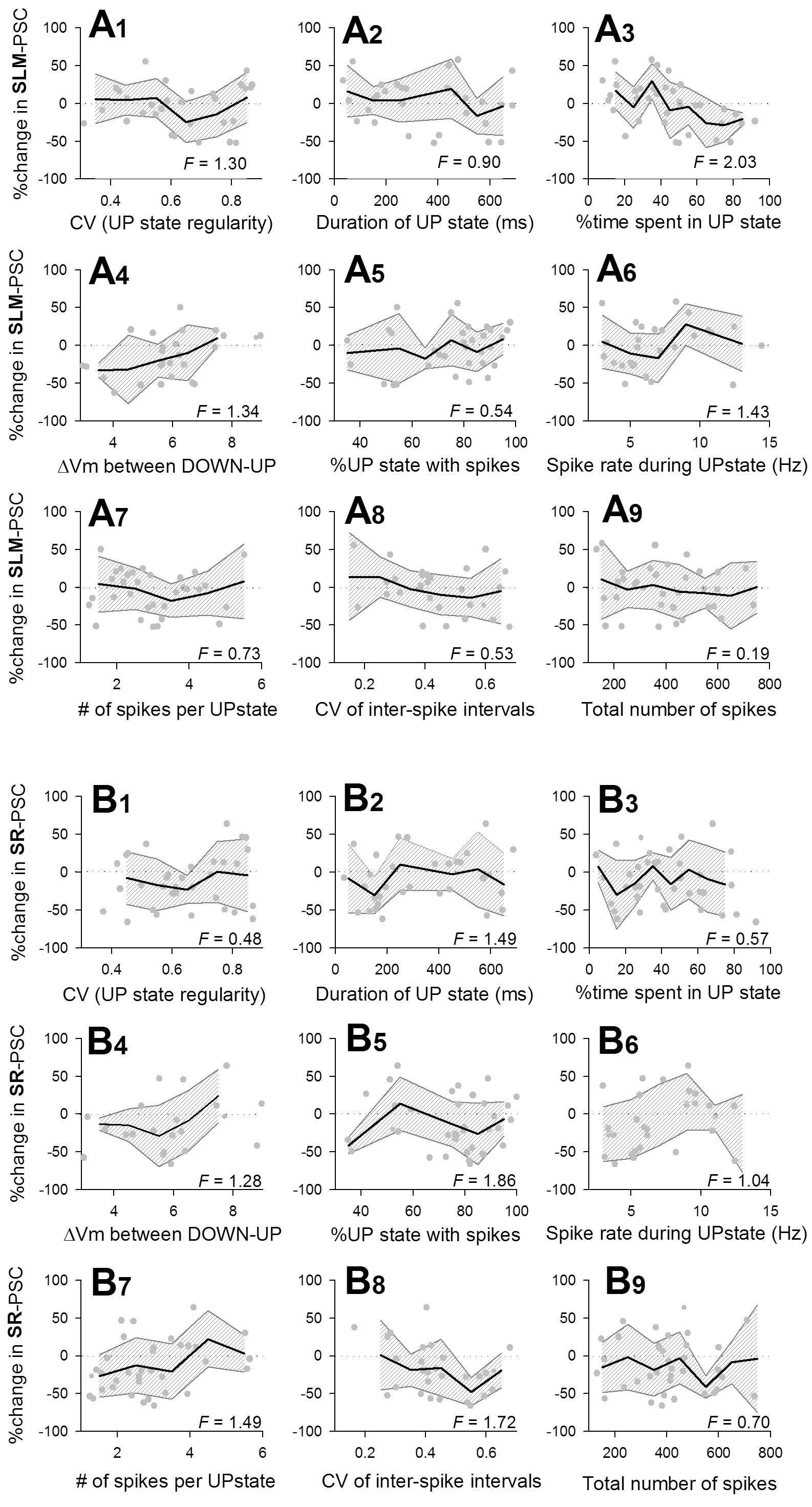

Supplement: Figure S2 — Relationships between synaptic plasticity and various parameters related to membrane oscillations. Changes in SLM-evoked (A) and SR-evoked (B) PSCs are plotted against (1) the regularity of slow oscillation rhythms (coefficient of variation in inter-UP state intervals), (2) the average time spent in single UP states, (3) the ratio of the total time spent for UP states to the total time recorded, (4) the voltage differences between UP and DOWN states, (5) the ratio of UP states accompanying spikes to the total UP states, (6) the mean firing rate, i.e., spikes per second, during UP states, (7) the mean number of spikes per UP state, (8) the coefficient of variation (CV) in inter-spike intervals, (9) and the total number of spikes during pACSF exposure. For each comparison, one-way ANOVA was performed to determine whether the parameter was correlated with the direction and magnitude of synaptic plasticity, but there was no statistical significance for any parameters tested. (0.58 MB JPG) [file pone.0001250.s002.jpg]
